# Ergosterol isolated from the basidiomycete *Pleurotus salmoneostramineus* affects *Trypanosoma cruzi* plasma membrane and mitochondria

**DOI:** 10.1186/s40409-017-0120-0

**Published:** 2017-05-30

**Authors:** Tatiana Rodrigues Alexandre, Marta Lopes Lima, Mariana Kolos Galuppo, Juliana Tonini Mesquita, Matilia Ana do Nascimento, Augusto Leonardo dos Santos, Patricia Sartorelli, Daniel Carvalho Pimenta, Andre Gustavo Tempone

**Affiliations:** 1 Center for Parasitology and Mycology, Instituto Adolfo Lutz, Avenida Dr. Arnaldo, 351, 8° andar, CEP 01246-000, São Paulo, SP Brazil; 2 Institute of Tropical Medicine of São Paulo, University of São Paulo (USP), São Paulo, SP Brazil; 3 Institute of Environmental, Chemical and Pharmaceutical Sciences, Federal University of São Paulo, São Paulo (UNIFESP), Diadema, SP Brazil; 4 Laboratory of Biochemistry and Biophysics, Butantan Institute, São Paulo, SP Brazil

**Keywords:** *Pleurotus salmoneostramineus*, Ergosterol, *Trypanosoma cruzi*, Mechanism of action

## Abstract

**Background:**

Major drawbacks of the available treatment against Chagas disease (American trypanosomiasis) include its toxicity and therapeutic inefficiency in the chronic phase of the infection, which makes it a concern among neglected diseases. Therefore, the discovery of alternative drugs for treating chronic Chagas disease requires immediate action. In this work, we evaluated the mushroom *Pleurotus salmoneostramineus* in the search for potential antiparasitic compounds.

**Methods:**

Fruit bodies of the basidiomycete *Pleurotus salmoneostramineus* were triturated and submitted to organic solvent extraction. After liquid-liquid partition of the crude extract, three fractions were obtained and the bioguided fractionation study was conducted to isolate the active metabolites. The elucidation of the chemical structure was performed using GC-MS and NMR techniques. The biological assays for antiparasitic activity were carried out using trypomastigotes of *Trypanosoma cruzi* and murine macrophages for mammalian cytotoxicity. The mechanism of action of the isolated compound used different fluorescent probes to evaluate the plasma membrane permeability, the potential of the mitochondrial membrane and the intracellular levels of reactive oxygen species (ROS).

**Results:**

The most abundant fraction showing the antiparasitic activity was isolated and chemically elucidated, confirming the presence of ergosterol. It showed anti-*Trypanosoma cruzi* activity against trypomastigotes, with an IC_50_ value of 51.3 μg/mL. The compound demonstrated no cytotoxicity against mammalian cells to the maximal tested concentration of 200 μg/mL. The mechanism of action of ergosterol in *Trypanosoma cruzi* trypomastigotes resulted in permeabilization of the plasma membrane, as well as depolarization of mitochondrial membrane potential, leading to parasite death. Nevertheless, no increase in ROS levels could be observed, suggesting damages to plasma membrane rather than an induction of oxidative stress in the parasite.

**Conclusions:**

The selection of naturally antiparasitic secondary metabolites in basidiomycetes, such as ergosterol, may provide potential scaffolds for drug design studies against neglected diseases.

**Electronic supplementary material:**

The online version of this article (doi:10.1186/s40409-017-0120-0) contains supplementary material, which is available to authorized users.

## Background

Chagas disease (CD) was described in 1909 by the Brazilian researcher Carlos Ribeiro Justiniano das Chagas [1]. It is currently estimated that 8 million people are infected worldwide, especially in 21 countries of Latin America. CD is responsible for 10,000 deaths annually, being a risk for 100 million people [2]. In Brazil, prevalent chronic cases of CD result from infections acquired in the past, affecting approximately three million individuals [2, 3]. However, in recent years, the occurrence of acute Chagas disease (ACD) has been observed in the Amazon, with the manifestation of isolated cases in other states [4]. *Trypanosoma cruzi* is the etiologic agent of CD, a hemoflagellate protozoan from the Kinetoplastida order [5]. Although CD has been described more than 100 years ago, the chemotherapy is, so far, limited to two nitro-heterocyclic drugs: benznidazole and nifurtimox [6]. Both are effective in the acute phase of the infection, with approximately 60–80% efficacy [7]. However, they are considered far from ideal in the chronic phase of the disease, with severe adverse effects and reduced efficacy [7, 8].

Due to this toxic and limited therapeutic arsenal, there is an urgent need to find new drugs. Considering that natural products have been providing interesting scaffolds for protozoan diseases, herein we investigated the potential of basidiomycetes, a rich source for antimicrobial compounds. For example, hypnofylline and panepoxidone isolated from the basidiomycete *Lentinus strigosus* and an ergosterol peroxide isolated from *Pleurotus ostreatus* have demonstrated anti-*T. cruzi* activities [9, 10]. Considering the genus *Pleurotus*, a number of secondary metabolites have been isolated with different biological properties, such as antitumor, antileishmanial, antimicrobial, anti-inflammatory and antitrypanosomal activities among others [10–14].

To the best of our knowledge, this is the first study that identified the antiparasitic potential of *Pleurotus salmoneostramineus* – popularly known as the “pink mushroom” – that grows on leaves of senescent plants found in Japan, New Guinea and Siberia. It is a popular mushroom because of the colorful body, flavor and texture, being rich in proteins, lipids, fiber, carbohydrates, vitamins and essential amino acids. It is generally used in reducing plasma cholesterol levels and prevents atherosclerosis [15, 16]. By using the bioguided fractionation, we isolated and chemically characterized the most abundant metabolite of *P. salmoneostramineus* with antitrypanosomal activity and evaluated its action on the plasma membrane, mitochondria and ROS levels of trypomastigote forms of the parasite.

## Methods

### Basidiomycete and chemicals

The basidiomycete *Pleurotus salmoneostramineus* was commercially purchased from Zucca Funzionale (Brazil) and its identification was confirmed by the Adolfo Lutz Institute. Phosphate-buffered saline (PBS), Roswell Park Memorial Institute Medium (RPMI 1640), Hank’s Balanced Salt Solution (HBSS), sodium azide, Triton X-100, carbonyl cyanide 4-(trifluoromethoxy)phenylhydrazone (FCCP), miltefosine, benznidazole (2-nitroimidazole), and the solvents dimethyl sulfoxide (DMSO), methanol (MeOH), hexane, ethyl acetate (EtOAc), butanol and dichloromethane were purchased from Sigma. The TLC Siligel 60 F254 aluminum chromatoplates with fluorescence indicators and common silica were purchased from Merk. Resazurin, sodium dodecyl sulfate (SDS), Sytox Green dye, Mitotracker Red CM-H2XROS, H_2_DCf-DA and MTT were purchased from Molecular Probes® (Invitrogen).

### Parasites, mammalian cells and experimental animals

Trypomastigotes of *Trypanossoma cruzi* (Y strain) were maintained in LLC-MK2 (ATCC CCL 7) cells using RPMI-1640 medium supplemented with 2% fetal bovine serum (FBS) at 37 °C in 5% CO_2_ humidified incubator. LLC-MK2 (ATCC) cells were maintained in RPMI-1640 medium without phenol red and supplemented with 10% SBF at the same conditions [17]. Peritoneal macrophages were collected from the peritoneal cavity of female BALB/c mice by washing with RPMI-1640 without phenol red, supplemented with 10% FBS. BALB/c mice were supplied by the animal breeding facility at the Adolfo Lutz Institute and maintained in sterilized cages under a controlled environment, receiving water and food ad libitum. Animal procedures were performed with the approval of the Research Ethics Commission (CEUA-IAL-Pasteur 01/2011), in agreement with the Guide for the Care and Use of Laboratory Animals from the National Academy of Sciences.

### Crude extract and liquid-liquid partition

Fruit-bodies of mushrooms basidiomycete *P. salmoneostramineus* (1000 g) were triturated and extracted with 1.4 L MeOH:H_2_O solution (1:1 v/v). This solution was sonicated (10 min), filtered (Whatman filter) and evaporated at 40 °C resulting in the crude extract. Subsequently, the crude extract was resuspended in 400 mL of water and partitioned using growing polarity solvents to afford the three fractions, n-hexane (518.8 mg), EtOAc (3432.9 mg) and butanol (1923.2 mg). Then, these fractions were dried at 40 °C and stored at –20 °C until analysis.

### Determination of 50% inhibitory concentration (IC_50_) against *Trypanosoma cruzi*

To determine the 50% inhibitory concentration (IC_50_) against free trypomastigotes of *T. cruzi* obtained from LLC-MK2 cultures, 1 × 10^6^ parasites/well were seeded on 96-well microplates. N-hexane, EtOAc and butanol fractions were dissolved in MeOH, serially diluted (two-fold) in RPMI-1640 medium and incubated with the parasites in a range concentration between 300 and 2.3 μg/mL (final volume 200 μL) for 24 h, 37 °C, 5% CO_2_. At the same conditions, the isolated compound was dissolved in DMSO, serially diluted (two-fold) and added to the highest concentration of 150 μg/mL. Benznidazole was used as a standard drug in a range concentration between 100 and 0.78 μg/mL. The parasite viability was determined by resazurin assay (0.11 mg/mL in PBS, 20 h incubation) using the FilterMax F5 Multi-Mode Microplate Reader, Molecular Devices (Sunnyvale, CA, USA), at 570 nm [18]. DMSO was used to dissolve the compounds and was included in the microplate at 0.5% (v/v) to avoid toxicity. Internal controls were also performed with DMSO. Additionally, the lethal effect of the isolated compound was confirmed on trypomastigotes using classical light microscopy analysis [19].

To determine the IC_50_ against intracellular amastigotes of *T. cruzi*, peritoneal macrophages were dispensed in 16-well chamber slide (NUNC, Thermo, USA) and maintained for 24 h in the same medium at 37 °C in a 5% CO_2_ humidified incubator for attachment. Non adherent cells were removed by two-step washings with medium. After 24 h, these cells were infected with 1 × 10^6^ culture trypomastigotes forms for 4 h. Subsequently, infected cells were incubated with the ergosterol in a range concentration between 100 and 0.78 μg/mL (final volume 200 μL) for 48 h. Finally, the slides were fixed with methanol, stained with Giemsa, and observed in a light microscope. The parasite load was defined by counting 400 macrophages/well by evaluating the number of infected macrophages. Benznidazole was used as standard drug in a range concentration between 50 and 0.39 μg/mL. DMSO was used at a maximal concentration of 0.5% (v/v) and incubated with cells as an internal control [20].

### Bioguided fractionation

The anti-trypomastigote activity guided the fractionation procedures. *n*-hexane, EtOAc and butanol fractions were subjected to thin layer chromatography (TLC) in aluminum plates of silica gel 60 F_254_ TLC with a fluorescent indicator at 254 nm. ^1^H and ^13^C RMN analysis, with spectra recorded at 300 and 75 MHz, were performed using a Bruker Ultrashield 300 Avance III spectrometer, respectively. CDCl3 (Aldrich) was used as the solvent with TMS as the internal standard. Chemical shifts (δ) are reported in ppm and the coupling constant (J) in Hz. *n*-hexane fraction were subjected to column chromatography (CC) with 89 g of silica gel G-60 (Merk, 0.063-0.200 mm) and 510 mg n-hexane fraction (CPS – column *Pleurotus salmoneostramineus*). Solvent system started with pure *n*-hexane (10:0, v/v) to pure EtOAc (0:10, v/v, 50 mL), followed by increasing amounts of MeOH (EtOAc 9:1, v/v to pure MeOH 0:10, v/v). New fractions obtained from this process (50 mL) were dried at 40 °C and stored at –20 °C until analysis. Structural elucidation of fraction CPS-3(3) were performed by analysis ^1^H and ^13^C RMN and GC-MS.

### Cytotoxicity against mammalian cells

To determine the 50% cytotoxic concentration (CC_50_) of the isolated compound, it was previously dissolved in DMSO, serially diluted (two-fold) in RPMI-1640 medium-10% SBF at maximum concentration of 200 μg/mL and incubated with 6 × 10^4^ peritoneal macrophages seeded on 96-well microplates, final volume of 200 μL during 48 h, 37 °C 5% CO_2_. Cell viability was determined by resazurin assay as above described. Same conditions were applied to determine the CC_50_ of the standard drug benznidazole, which was tested in a range concentration between 200 and 1.56 μg/mL [21]. DMSO was used to dissolve the compounds and was included in the microplate at 0.5% (v/v) to avoid toxicity. Internal controls were also performed with DMSO.

### Hemolytic activity

The hemolytic activity of the isolated ergosterol was evaluated in BALB/c erythrocytes [22]. A 3% suspension of mouse erythrocytes was incubated for 2 h with the isolated compound at 100 μg/mL in 96-well U-shape microplate at 25 °C and the supernatant was read at 550 nm in a spectrophotometer FilterMax F5 Multi-Mode Microplate Reader, Molecular Devices (USA). Ultrapure distilled water was used as a positive control (100% hemolysis) and phosphate-buffered saline (PBS) as a negative control (0% hemolysis). DMSO was also used at 0.5% as internal control.

### Mechanism of action of ergosterol

As standard conditions, free trypomastigotes of *T. cruzi* obtained from LLC-MK2 cultures were washed twice and seeded on 2 × 10^6^/well in PBS to 96-well black polystyrene microplate. The isolated compound was tested at the IC_99_ value (100 μg/mL) and incubation was performed at 37 °C, 5% CO_2_. Fluorescence was monitored using FilterMax F5 Multi-Mode Microplate Reader, Molecular Devices (USA) at respective wavelengths. In all mechanism assays, the following internal controls were used in presence of respective dyes (Sytox Green, MitoTracker Red CM-H2XROS or H_2_DCf-DA): the background fluorescence of the isolated compound at the respective wavelengths; the possible interference of DMSO; untreated (control) trypomastigotes; and medium without any cells. Samples were tested in triplicate and at least three independent assays were performed.

### Evaluation of plasma membrane permeability

Under standard conditions, trypomastigotes were incubated with 1 μM Sytox Green for 15 min at 37 °C, 5% CO_2_ in the dark [23]. Then, the isolated compound was added at 100 μg/mL and the fluorescence was monitored every 20 min during 80 min at excitation (λex) and emission (λem) wavelengths of 485 and 520 nm, respectively. The maximum membrane permeabilization was obtained with 0.5% Triton X-100, as a positive control.

### Evaluation of the mitochondrial membrane potential

Under standard conditions, trypomastigotes were incubated with the isolated compound at 100 μg/mL during 60 min and then MitoTracker Red CM-H2XROS (500 nM) was added and incubated for 40 min in dark. The minimal mitochondrial membrane potential was obtained by treatment of trypomastigotes with 10 μM FCCP, a known mitochondrial uncoupler. Trypomastigotes were washed twice with PBS before fluorescence reading at λex = 540 nm and λex = 595 nm [21].

### Detection of reactive oxygen species (ROS)

Intracellular ROS levels were measured using fluorescent probe H_2_DCf-DA. Under standard conditions, except by the use of HBSS solution instead of PBS, trypomastigotes were incubated with the isolated compound ergosterol at 100 μg/mL and sodium azide (10 mM) as a positive control in order to obtain high levels of ROS production during 60 min. Then, fluorescent probe H_2_DCf-DA was added (5 μM, 15 min) and the fluorescence intensity measured at λex = 485 nm and λex = 520 nm [23].

### Statistical analysis

The IC_50_ and CC_50_ values were calculated using sigmoid dose-response curves in GraphPad Prism 5.0 software, and the 95% confidence intervals are included in parentheses. The ANOVA test was used for significance p value. The data obtained from the mechanism of action represent the mean and standard deviation (SD) of triplicate samples from at least two independent assays.

## Results

### Bioguided fractionation and antitrypanosomal activity

The three obtained fractions (n-hexane, EtOAc and butanol) were incubated with free trypanosomes during 24 ho and the viability determined by the resazurin assay. All fractions demonstrated anti-trypomastigote activity against the parasite resulting in 100% of death at the highest concentration of 300 μg/mL. The n-hexane, EtOAc and butanol fractions showed IC_50_ values of 7.9 μg/mL, 28.0 μg/mL and 58.3 μg/mL, respectively (Table 1). The TLC analysis determined a good resolution for the mixture of n-hexane:EtOAc (1:1, v/v) (data not shown). Together, the analytical assays and the anti-trypomastigote activity of n-hexane fraction guided the subsequent separations, yielding 17 new fractions, which were compiled into ten groups (CPS1-10) based on their chromatographic similarities. The antitrypanosomal activity was detected in a crystallized fraction [namely CPS-3(3)], which resulted in an IC_50_ value of 51.3 μg/mL (46.1–57.0) against the trypomastigotes (Table 1).

**Table 1:** Antitrypanosomal activity of ergosterol and cytotoxicity against mammalian cells

Compound	IC_50_ (μg/mL) 95% CI trypomastigote	IC_50_ (μg/mL) 95% CI amastigote	CC_50_ (μg/mL) 95% CI macrophages	Selectivity index	Hemolytic activity (SD)
Ergosterol	51.3 (46.0–57.0)	>100	> 200	> 3.9	7.03% (±11.6)
Benznidazole	4.4 (3.7–5.1)	1.9 (1.2–2.3)	> 200	> 45.0	nd

IC_50_: 50% effective concentration; CC_50_: 50% cytotoxic concentration; 95% CI: 95% confidence interval; SI: selectivity index calculated as (SI = CC_50_ mammalian cell/IC_50_ trypomastigotes); nd: not determined

### Biological activity of ergosterol

The viability of trypamastigotes of *T. cruzi* and mammalian cells treated with ergosterol was determined by resazurin and MTT assay after 48 h of incubation. Ergosterol showed a moderate anti-trypomastigote activity with IC_50_ = 51.3 μg/mL; benznidazole was used as standard drug and resulted in an IC_50_ = 4.4 μg/mL. Additionally, light microscopy analysis confirmed the lethal effect of ergosterol in trypomastigotes. Ergosterol was also tested against the intracellular amastigotes of *T. cruzi*, but demonstrated no activity. Ergosterol (and benznidazole) showed lack of toxicity to BALB/c peritoneal macrophages to the maximal tested concentration of 200 μg/mL (Table 1). The hemolytic activity of ergosterol was tested in mice erythrocytes, but the compound induced no significant hemolysis (7.03% ± 11.6) to the highest tested concentration (Table 1) when compared to untreated cells.

### Ergosterol structural elucidation

The CPS-3(3) fraction was subjected to ^1^H and ^13^C RMN and GC-EI-MS analysis for structural elucidation. ^13^C RMN spectra reveals C_28_-sterol ergostane skeleton, including signals of six unsaturated carbonsat δ_C_ 116.3-141.4 corresponding to C-5 (δ_C_ 139.8); C-6 (δ_C_ 119.6), C-7 (δ_C_ 116.3), C-8 (δ_C_ 141.4), C22 (δ_C_ 135.6) and C23 (δ_C_ 131.9). Methyl carbons were observed in C-18 (δ_C_ 12.1), C-19 (δ_C_ 16.3), C-21 (δ_C_ 21.1) C-26 (δ_C_ 28.3), C-27 (δ_C_ 19.7) and C-28 (δ_C_ 17.6), whereas hydroxyl group was observed in C-3 (δ_C_ 70.5). ^1^H RMN corroborated sterol Δ^5,7^ structure by signals δ_H_ 5.58 (*dd*, J = 3.0; 5.5 Hz) and 5.38 (*dd*, J = 2.9; 5.4 Hz) diagnostic for olefin hydrogens H-6 and H-7, besides multiplet in δ_H_ 3.64 (H-3) indicate the presence of hydrogen linked to carbinolic carbon. Double bonds were observed at signal 5.20 (m) relative to H-22 and H-23. Still, signals at region δ_H_ 0.8 and 1.1 relative to methyl groups identified two singlet hydrogen in δ_H_ 0.95 (CH_3_-C-18) and 0.65 (CH_3_-C-19), and four duplets in δH 0.82 (CH3-27), 0.84 (CH3-26); 0.92 (CH3-28), 1.04 (CH3-21). The assignment of all the carbon signals was performed by comparison with the reported data (Table 2). Table 1 compiles 1H and 13C RMN comparing with literature [24, 25].

**Table 2:** ^13^C and ^1^H RMN data for ergosterol (75 MHz and 300 MHz)

Position	δ_C_ EXP	δ_C_ LIT.^a^	δ_H_ EXP	δ_H_ LIT.^b^
1	38.4	38.5		
2	31.9	32.1		
3	70.5	70.5	3.64 m (1H)	3.61 m (1H)
4	40.8	40.9		
5	139.8	139.8		
6	119.6	119.7	5.58 dd (5.5, 3.0 Hz, 1H)	5.56 dd (5.4, 2.2 Hz, 1H)
7	116.3	116.4	5.38 dd (5.4, 2.9 Hz, 1H)	5.38 dd (5.4, 2.5 Hz, 1H)
8	141.4	141.3		
9	46.2	42.3		
10	37.1	37.1		
11	21.1	21.1		
12	39.1	39.1		
13	42.9	42.9		
14	54.6	54.6		
15	22.9	23.1		
16	28.3	28.3		
17	55.7	55.8		
18	12.1	12.1	0.95 s (3H)	0.95 s (3H)
19	16.3	16.3	0.65 s (3H)	0.63 s (3H)
20	40.3	40.4		
21	21.1	21.2	1.04 d (J = 6.6 Hz, 3H)	1.00 d (J = 6.6 Hz, 3H)
22	135.6	135.6	5.20 m (1H)	5.20 m (1H)
23	131.9	132.1	5.21 m (1H)	5.20 m (1H)
24	42.9	42.9		
25	33.1	33.1		
26	19.9	20.0	0.84 d (J = 6.7 Hz, 3H)	0.84 d (J = 6.7 Hz, 3H)
27	19.7	19.7	0.82 d (J = 6.7 Hz, 3H)	0.83 d (J = 6.7 Hz, 3H)
28	17.6	17.6	0.92 d (J = 6.6 Hz, 3H)	0.95 d (3H)

^a^ [21]

^b^ [22]

**Fig. 1:**
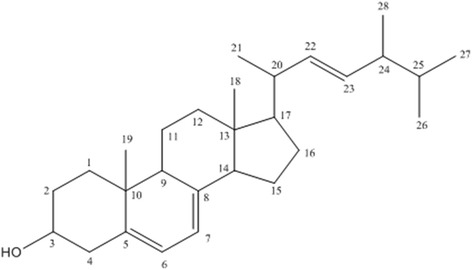
Structure of ergosterol confirmed by ^13^C, ^1^H RMN and GC-EI-MS

GC-EI-MS also confirmed the identity and purity of ergosterol (Fig. 1) by the presence of m/z 396 corresponding to molecular formula C_28_H_44_O and fragments m/z 378 [M–H_2_O]^+^, m/z 363 [M–CH_3_-H_2_O]^+^, m/z 253 [M–side chain-H_2_O]^+^ and m/z 271 [M–side chain]^+^, a typical fragmentation profile of sterols [26] (Additional file 1).

### Permeabilization of plasma membrane

**Fig. 2:**
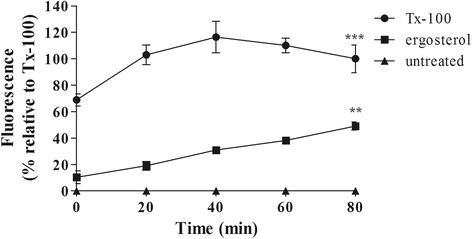
Effect of ergosterol on plasma membrane permeabilization of trypomastigotes of *T. cruzi*. Sytox Green (1 μM) fluorescence was measured spectrofluorimetrically every 20 min. Minimum and maximum permeabilization were obtained by non-treatment and Tx-100 0.5%, respectively. Fluorescence was quantified by calculating the mean percentages of untreated (0%) and Tx-100-treated (100%) trypomastigotes. *** *p* < 0.001 and **p < 0.002. A representative assay is shown

Ergosterol (at IC_99_ 100 μg/mL) was incubated for 80 min with trypomastigotes and the permeability of plasma membrane was evaluated through the fluorescence of Sytox Green dye (1 μM). The entrance of Sytox Green dye is allowed solely in injured plasma membrane, otherwise, its influx is precluded and no fluorescence can be measured. The increase of the dye caused by ergosterol (Fig. 2) were significant (*p* < 0.002) compared to untreated parasites, suggesting the alteration of the plasma membrane permeability when compared to the positive control with 0.5% Tx100 (*p* < 0.001), a non-ionic detergent (Fig. 2).

### Alteration of mitochondrial membrane potential

**Fig. 3:**
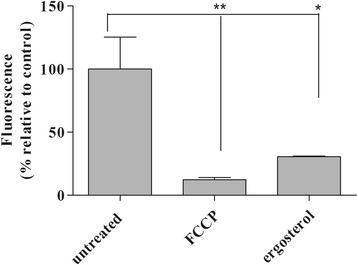
Effect of ergosterol in the mitochondrial function of trypomastigotes. Fluorescence of Mitotracker Red CM-H2XROS dye (500 nM) was spectrofluorimetrically measured after 60 min of incubation. Maximal and minimal fluorescence was achieved by non- or FCCP (10 μM) treatments, respectively. Fluorescence was quantified by calculating the mean percentage of untreated parasites (100%). *p < 0.001. A representative assay is shown

Alteration in mitochondrial membrane potential was determined by the fluorescence of Mitotracker Red CM-H_2_XROS dye (500 nM) in trypomastigotes treated with ergosterol (100 μg/mL) for 60 min. Ergosterol treatment caused significant decreased in the accumulation of Mitotracker Red at mitochondria indicating an effect of depolarization since normal potential guided maximal accumulation of dye in untreated trypomastigotes (control). Mitochondrial dysfunction promoted by ergosterol was comparable to that achieved by the treatment with 10 μM FCCP (Fig. 3).

### Reactive oxygen species (ROS) production

**Fig. 4:**
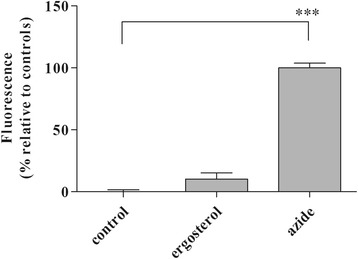
Effect of ergosterol in the ROS production of trypomastigotes. Fluorescence of H_2_DCF-DA was measured after 60 min of incubation. Fluorescence was quantified by calculating the mean percentage of untreated (0%) and sodium azide-treated (100%) trypomastigotes. ***(*p* < 0.001). A representative assay is shown

The production of ROS in trypomastigotes incubated with ergosterol (60 min, 100 μg/mL) was evaluated by the fluorescence of H_2_DCF-DA dye (5 μM). Trypomastigotes treated with ergosterol showed no significant increase in the production of ROS, showing levels similar to the untreated parasites. Trypomastigotes treated with sodium azide (10 mM) showed intense fluorescence as a positive control (Fig. 4).

## Discussion

Natural secondary metabolites isolated from basidiomycetes have been described as rich source of bioactive molecules. Terpenoids isolated from *Lentinus strigosus* and lanostane-type triterpenes isolated from *Astraeus hygrometricus* are some examples of compounds with promising activity against protozoan parasites such as *T. cruzi* and *Leishmania* [9, 24, 27].

Few studies on the chemical composition of mushrooms *Pleurotus salmoneostramineus* have been performed whereas the evaluation of their biological activities remains underexplored [15, 28, 29]. In the present study, the fractionating of the organic crude extract of *P. salmoneostramineus* yielded an antitrypanosomal substance. Our initial data demonstrated the presence of antiparasitic compounds in fractions of different polarities, such as *n*-hexane, EtOAc and butanol, which suggests that no single compound exists with efficacy against *T. cruzi* parasites. Among them, the ergosterol was isolated in large amount as a crystalized pure substance further elucidated by NMR and GC-EI-MS based in the literature data [24–26].

Ergosterol is the major sterol that composes membranes of *T. cruzi* parasites – including plasma, inner mitochondrial and glycosomal membranes – instead of cholesterol, its counterpart in mammalian cells that is usually confined to the plasma membrane [30–32]. *Trypanosoma cruzi* normally synthesizes sterols and occasionally leucine from carbon precursors [33]. The singularity of the sterol composition of *T. cruzi* parasites – closest to fungi cells than to mammalian host cells – has validated SBP as target for new therapeutic drugs [34]. Indeed, *T. cruzi* CPY51 (*Tc*CPY51) inhibitors – posaconazole and ravuconazole – entered in Drugs for Neglected Diseases Initiative (DNDi) clinical trials for Chagas disease despite the clinical failure recently reported [35, 36]. In general, the efficacy of *Tc*CYP51 inhibitor has been considerably variable depending on the parasite strain and time of exposure [36, 37].

In addition to *Tc*CYP51 inhibitors, sterols isolated from different natural sources including plants, marine sponges and amphibians have demonstrated to present antiprotozoan activities. Interestingly, ergosterol itself can be trypanocidal at unusual levels from its natural metabolism [22, 38, 39]. For example, *T. cruzi* amastigotes had 94% of growth inhibited by 10 μM of ergosterol [40]. A previous study demonstrated that ergosterol peroxide, but not ergosterol, isolated from the basidiomycete *Pleurotus ostreatus* also presented anti-*T. cruzi* activity, with an IC_50_ value of 6.7 μg/mL against the insect form, epimastigotes [10]. When compared to our results, the previous work suggests that ergosterol peroxide is approximately 8-fold more active than ergosterol against trypomastigote forms. This could be due to the presence of the endoperoxide group, a known structure presenting potential biological properties [41, 42].

Additionally, differences may rely on metabolic specificities among forms of *T. cruzi*. For example, extracellular epimastigotes found in the insect vector have been described to be more susceptible to drugs than trypomastigotes, a parasite form found inside cells and bloodstream of vertebrate hosts [43]. Relapse and therapeutic failure have been described to arise from resident trypomastigotes, usually not affected by conventional therapy, which preferentially target intracellular amastigotes [44]. This information highlight the importance of selecting new anti-trypomastigote compounds.

The lethal action of ergosterol against trypomastigotes of *T. cruzi* was investigated. Ergosterol-treated trypomastigotes showed a rapid plasma membrane permeabilization as determined by the Sytox Green fluorescence. Alterations in plasma membrane composition are known to modify fluidity and cellular morphology. For example, high concentrations of ergosterol have been speculated to be associated with rigidity, whereas low concentrations cause disorganization/disruption of the plasma membrane [45–47]. In our work, the physicochemical properties of ergosterol may have contributed to its crossing through plasma membrane, thus rapidly altering the permeability by disturbing normal lipid composition and modifying its fluidity and permeability. Disturbances of plasma membrane permeability largely contribute to trypomastigote death. Numerous metabolites from natural origin including soulamarin, dermaseptins and phylloseptins have been reported to trigger such death mechanism [20, 23]. Moreover, drugs in clinical use for leishmaniasis such as miltefosine and amphotericin B are known to alter the permeability of plasma membrane [48, 49]. In this study, *T. cruzi* trypomastigotes treated with ergosterol suffered a rapid depolarization of the mitochondrial membrane potential probably by a direct accumulation in the mitochondria or as a secondary effect of the disturbance in plasma membrane permeability. Corroborating the previous hypothesis, exogenous ergosterol, but not cholesterol, is capable of abolishing the ketoconazole-induced massive swelling of the mitochondria [50].

Under conditions of permeability disturbance and loss of mitochondrial potential, the single mitochondria of *T. cruzi* may produce excessive ROS [51–53]. Our results demonstrated that ergosterol-treated trypomastigotes did not present significant alteration in ROS levels. These data suggest that exogenous supplementation of ergosterol may target the mitochondria and act as ROS scavengers, as indicated by a recent finding reported elsewhere [46]. Additionally, *Leishmania (Leishmania) donovani* parasites that are CYP51-defective constitutively present low ergosterol levels and higher susceptibility to oxidative stress induced by antimony [54]. The cell viability is increased during exposure to antimony by in vitro supplementation of ergosterol [45]. Therefore, we suggest that oxidative stress may not be contribute to the mechanism of action of ergosterol in *T. cruzi* parasites.

## Conclusion

The basidiomycete mushroom *Pleurotus salmoneostramineus* demonstrated to be an interesting and underexplored natural source for antiparasitic metabolites. Disturbances in the permeability of plasma membrane and loss of mitochondrial membrane potential without involvement of oxidative stress were pointed as initial mechanisms of action of ergosterol against *T. cruzi*. Although ergosterol is a constituent of the plasma membrane of *T. cruzi*, it is also effective to eliminate the parasite. The compound is a low cytotoxic substance that may be useful as scaffold for future synthesis of new derivatives against *Trypanosoma cruzi*.

## Additional file

Additional file 1: Ergosterol fragmentation proposal interpreted from GM-EI-MS analysis. (DOCX 31 kb)

## Abbreviations

ACD: Acute Chagas disease
CC: Column chromatography
CC_50_: 50% cytotoxic concentration
CD: Chagas disease
CPS: Column *Pleurotus salmoneostramineus*
DMSO: Dimethyl sulfoxide
DNDi: Drugs for Neglected Diseases Initiative
EtOAc: Ethyl acetate
FBS: Fetal bovine serum
HBSS: Hank’s Balanced Salt Solution
IC_50_: 50% inhibitory concentration
PBS: Phosphate-buffered saline
ROS: Reactive oxygen species
RPMI: Roswell Park Memorial Institute Medium
SBP: Sterol biosynthetic pathway
SDS: Sodium dodecyl sulfate
*Tc*CPY51: *T. cruzi* CPY51
TLC: Thin layer chromatography
